# (Not) being granted the right to belong—Amateur football clubs in Germany

**DOI:** 10.1177/10126902211061303

**Published:** 2021-12-08

**Authors:** Tina Nobis, Carlos Gomez-Gonzalez, Cornel Nesseler, Helmut Dietl

**Affiliations:** 9373 Humboldt-Universität zu Berlin, Germany; 27217 University of Zurich, Switzerland; 8018 Norwegian University of Science and Technology, Norway; 27217 University of Zurich, Switzerland

**Keywords:** amateur football, belonging, migration, sports clubs, field experiment, discrimination

## Abstract

Empirical studies show that first- and second-generation immigrants are less likely to be members of sports clubs than their non-immigrant peers. Common explanations are cultural differences and socioeconomic disadvantages. However, lower participation rates in amateur sport could be at least partly due to ethnic discrimination. Are minority ethnic groups granted the same right to belong as their non-immigrant peers? To answer this question, this paper uses publicly available data from a field experiment in which mock applications were sent out to over 1,600 football clubs in Germany. Having a foreign-sounding name significantly reduces the likelihood of being invited to participate. The paper concludes that amateur football clubs are not as permeable as they are often perceived to be. It claims that traditional explanations for lower participation rates of immigrants need to be revisited.

## Introduction

As this article focusses on the topic of sport in immigrant societies, it touches upon a subject that has been well researched within the past two decades. Numerous sport sociological works focus on first- and second-generation immigrants, on other specific immigrant groups (e.g. refugees) or on “minority ethnic groups” – a term which is often employed to refer to individuals who do not share a given or an ascribed attribute (e.g. religion, race, citizenship) with the majority population. With specific regard to the European discourse, the narrative usually follows the thread of what Coakley (2015) has called the great sport myth and with which he describes the “pervasive and nearly unshakable belief in the inherent purity and goodness of sport” (p. 403). Research about sport in immigrant societies often starts with the assumption that sports clubs have considerable potential for integration because they are formally open to everybody. Thus, sports cubs could, potentially, offer good grounds for common activities for all population groups. These assumptions often refer to theories of integration and lead to empirical surveys about who participates in sports clubs and how members can benefit from sport activities (Adler Zwahlen et al., 2018; Makarova and Herzog, 2014; Seippel, 2005; Smith et al., 2019; Spaaij and Broerse, 2019; Stura, 2019; Theeboom et al., 2012; Walseth, 2006; Walseth and Fasting, 2004).

Empirical studies of participation in sports clubs, however, overwhelmingly show that first- and second-generation immigrants, people from refugee backgrounds and other marginalized groups (e.g. Black and minority ethnic groups) are less likely to be members than their peers (Elling and Claringbould, 2005; Feiler and Breuer, 2020; Higgins and Dale, 2013; Makarova and Herzog, 2014; Nielsen et al., 2013; van Haaften, 2019). While these findings might raise questions about potential discrimination in sport and thus conquer the great sport myth or the assumption about the integrative potential of sport, they seldom do so. Instead, common explanations for the lower participation rates of immigrants and minority ethnic groups include cultural differences, socioeconomic disadvantages, different leisure preferences, and self-exclusion (Burrmann et al., 2017; Higgins and Dale, 2013; Kleindienst-Cachay, 2007; Mutz and Burrmann, 2015; Nielsen et al., 2013; Spaaij, 2012; van Haaften, 2019).

Even though the most common theme in recent publications refers to integration or inclusion, we are not suggesting that exclusion, discrimination, and racism in sport have not been researched at all (for a detailed literatue review, see Spaaij et al., 2019; for a thorough discussion of the integration theme, see Agergaard, 2018). Several studies that focus on different minorities demonstrate that people of color, people from minority backgrounds and individuals of African origin are underrepresented in leading positions of sports organisations in European countries and the U.S. (Bradbury, 2013; Heim et al., 2021; Hylton, 2018; Lapchick, 2021). Qualitative studies show that sport can “expose participants to social exclusion, racism and cultural resistance” (Spaaij, 2015: 304) and that refugees, Black athletes and minority ethnic groups may experience discrimination, microaggressions, othering, or assimilation pressure in sports clubs (Burdsey, 2011; Engh et al., 2017; Massao and Fasting, 2014; Spaaij, 2012). Furthermore, some publications focus on how a sports club's culture can evoke the exclusion of minorities (Michelini et al., 2018; Seiberth, 2012). However, we mostly find qualitative studies that concentrate on experiences of discrimination after minority groups have already joined a sports club. If and how participation rates in sports clubs are affected by discrimination and exclusion has not been studied in detail; consequently, empirical data illustrating how access to sports clubs can be denied is still missing.

In this article, access to sports clubs is analysed from the perspective of exclusion. Instead of asking why minority ethnic groups do not wish to participate in recreational sports clubs and instead of using sports club membership as an indicator for integration, the authors ask if immigrants who wish to participate are being granted the right to do so as non-immigrants. To that end, this paper refers to the theoretical concept of belonging. Other than theories of integration, this framework can help to understand that the lower sport participation of immigrants does not necessarily point to integration deficits amongst immigrants but that it can also be regarded as a matter of not being granted the right to belong by sports clubs.

Consequently, this paper applies a different methodological approach than the one that has often been used in the past. Instead of using survey data, we will use publicly available data from a field experiment approach in which individuals with foreign-sounding names stated a desire to join an amateur football club (Gomez-Gonzalez et al., 2021; Nesseler et al., 2019). With this approach, we can test causal relationships between being invited to a training session and signing the respective e-mail with a foreign-sounding name. Similar designs have been used to demonstrate that religious minorities and those who are perceived as foreign face discrimination when trying to access domains like the labour market (Bertrand and Mullainathan, 2004; Quillian et al., 2017; Riach and Rich, 2003; Thijssen et al., 2021; Zschirnt and Ruedin, 2016), housing (Auspurg et al., 2019; Diehl et al., 2013; Sawert, 2020), shopping (Bourabain and Verhaeghe, 2019), car riding (Liebe and Beyer, 2021), and the sharing economy (Edelman et al., 2017). However, the field of sport has not been deeply investigated in this way.

This paper focuses on one country—Germany. The German case is of specific interest with regard to the question addressed in this article. First, Germany can be described as an immigrant society, as approximately 26% of the population are first- or second-generation immigrants (Fachkommission der Bundesregierung zu den Rahmenbedingungen der Integrationsfähigkeit, 2020). Second, it is a country in which sports clubs are a relevant setting for sport activities, as about 27 million people are registered in approximately 88,000 sport clubs (Deutscher Olympischer Sportbund, 2020). Furthermore, content analyses have shown that the German discourse usually follows the assumption that sports clubs bear integrative potentials for immigrants, whereas discrimination and exclusion in sport remains a highly understudied topic (Nobis and El-Kayed, accepted). Interestingly, research has also shown that immigrants are less likely to be members of a sports club on the one hand, but that this does not hold true for male adolescents on the other hand. Reliable data for adolescents shows that male immigrants participate at equal levels in sports clubs as male non-immigrants (Nobis and El-Kayed, 2019). This is of specific interest for this article. If the data of the field experiment shows that male immigrants experience discrimination when trying to access a sports club, it also raises the question of whether equal participation rates can and should be regarded as an indicator for the absence of discrimination and inequality in future research (Elling and Claringbould, 2005).

The remainder of this paper is organized as follows. The next section introduces the concept of “belonging” to explain theoretically how access to a club can be granted or denied. As we use data from a field experiment performed by Gomez-Gonzalez et al. (2021), we describe the research design and methods of the study, and present the empirical findings. They show that individuals with foreign-sounding names are not granted the same rights to belong to a sports club as individuals with German-sounding names. Finally, we discuss the results and conclude the paper.

### Sports clubs and the politics of belonging

This article does not use integration as a theoretical frame when addressing the topic of sport in immigrant societies. We do not frame membership in sports clubs as an indicator of integration or ask how well immigrants have assimilated to mainstream sports culture. Rather, we approach the topic from a different theoretical perspective. We use the concept of belonging. This is especially helpful in understanding the logic and processes of inclusion and of exclusion in clubs. It shows how the formal openness of associations can be restricted by certain politics of belonging.

Amateur sports clubs can be defined as voluntary associations that are part of the “third sector.” The third sector differs from the state (first) and market (second) sectors, as well as from the informal, private sphere. Like other voluntary associations—but unlike organizations in the market sector—amateur sports clubs have a non-profit constraint. They rely on the principle of open and voluntary membership, meaning that everyone can become a member, but no one is obliged to do so (unlike state institutions such as schools). Amateur sports clubs pursue the goal of producing and providing “goods”—namely, sport offerings—for which participants usually pay a membership fee. Sports clubs are often described as “prosumer” organizations: relying on the principle of democratic self-organization, members voluntary engage to provide club goods (Baur and Braun, 2003; Etzioni, 1973; Heinemann and Horch, 1988).

In Germany, amateur sports clubs are the most popular voluntary association. According to the German Olympic Sport Federation, some 27 million people are registered in 88,000 clubs, of which more than 24,000 are devoted to football (Deutscher Olympischer Sportbund, 2020). However, empirical studies show that members of sports clubs do not come equally from all parts of the population. Women, older adults and low wage earners are less likely to be members of clubs or volunteers (Hartmann-Tews, 2006; Haut and Emrich, 2011; Nobis and El-Kayed, 2019; Wicker et al., 2020). Furthermore, the following findings are often cited: (a) the underrepresentation of first- and second-generation immigrants in sports clubs is more prevalent in female than in male sports; (b) immigrant male adolescents report membership in clubs just as much as their non-immigrants peers; (c) participation rates of first- and second-generation immigrants are higher in football and martial arts than in other sports; and (d) other sport activities (e.g. extra-curricular activities at school, fitness studios, informal settings) are less selective than clubs. (e) Additionally, recent research shows that male adolescent immigrants with a Turkish background are more likely to be a member of sports clubs than their non-immigrant peers. However, male adolescents with a Polish background are slightly underrepresented. Older data suggests that male adolescents with an Italian background are equally involved in sports clubs as male non-immigrants (Feiler and Breuer, 2020; Fussan and Nobis, 2007; Mutz and Burrmann, 2015).

The lower sport participation rate of immigrants is normally framed as a matter of “social integration”; indeed, many academics focus on cultural differences to explain differences in participation (Nobis and El-Kayed, accepted). To this we raise the following challenge: What if lower participation rates of immigrants tell us less about their integration deficits, and more about discrimination against them, such that they are excluded from clubs?

#### Belonging and the politics of belonging

As mentioned earlier, a useful theoretical construct here is the concept of *belonging.* Nira Yuval-Davis (2006, 2011) in particular has pointed out that “it is important to differentiate between *belonging* and the *politics* of belonging” (Yuval-Davis, 2011: 10) on an analytical level.

Belonging describes the dynamic emotional attachment with social and/or geographical locations. It is finding a space of “familiarity, comfort, security, and emotional attachment” (Antonsich, 2010: 464; see also Yuval-Davis, 2006). Belonging is multidimensional, as individuals can belong to different social locations that may change over time. Gender, class, nation, and kinship can be reference points of belonging. Equally, clubs, associations, families, and even street gangs can be reference points (Pfaff-Czarnecka, 2013; Yuval-Davis, 2006).

In today's world, (1) people can simultaneously belong to two or more countries; they can combine different professions or even religions; (2) they can change belonging while going through different stages in life—changing age groups and passing through different stages of status. (3) There is a situational multiplicity—when people divide their time between home, school, friends, hobby club, or religious organisation. (4) There are also diverse horizons of belonging: family, ethnic group, nation-state, and the world—and these horizons can coexist in a mode full of tensions (Pfaff-Czarnecka, 2013: 22).

How do individuals develop a sense of belonging? Pfaff-Czarnecka (2013) suggests that belonging is experienced through “identification, embeddedness, connectedness and attachments” (p. 13). Hage (2002) understands belonging as the “combined result of trust, feeling safe, community, and the sense of possibility” (cited by Pfaff-Czarnecka, 2013: 13). Yuval-Davis (2006) claims that belonging is constructed on three levels: social locations, emotional attachments, and ethical and political values. According to Mecheril (2018), belonging comprises three elements: membership, efficacy, and attachment. *Membership* refers to formal regulations about who belongs and who does not (e.g. citizenship or residence permits) and to informal practices of being recognized as a member by significant others. *Efficacy* refers to the possibility of participating effectively in a social entity. *Attachment* encompasses emotional bonding, moral obligations, familiarity, and connectedness.

While some authors primarily focus on the micro-level of belonging (Pfaff-Czarnecka, 2013), others point out that analyses should equally consider how individuals are granted the *right* to belong (Antonsich, 2010; Wood and Waite, 2011; Yuval-Davis, 2006). Belonging is not just a matter of an individual's choice, but is strongly related to being recognized and understood (Wood and Waite, 2011). Consequently, belonging should be analysed both as a “personal, intimate, feeling of being ‘at home’ in a place (place-belongingness) and as a discursive resource that constructs, claims, justifies, or resists forms of socio-spatial inclusion/exclusion (politics of belonging)” (Antonsich, 2010: 644). Such a multilevel approach—embodying Nira Yuval-Davis's distinction between belonging and the politics of belonging—considers practices of inclusion/exclusion simultaneously on a micro- and on a meso/macro-level. It thereby avoids the trap of what Antonsich (2010) describes as either a “socially de-contextualized individualism or an all-encompassing social(izing) discourse” (p. 644).

The politics of belonging involve the construction and the maintenance of boundaries by hegemonic powers, as well as “the inclusion or exclusion of particular people, social categories and groupings within these boundaries by those who have the power to do this” (Yuval-Davis, 2011: 18). Crowley (1999: 30) referred to the politics of belonging as the “dirty work” of boundary maintenance. Using the metaphor of a night club where many queue up but only a few are granted entry, Crowley pointed out that the politics of belonging are a matter of boundary-making and of separating us from them (Yuval-Davis, 2006). Yuval-Davis’s (2006) reference to Crowley underlines her point that the politics of belonging also include struggles about what is required from a person to belong (Lenneis and Agergaard, 2018). However, the requirements for belonging can constitute more or less permeable boundaries. Common descent is probably the most racialized and least permeable requisite, whereas “using a common set of values, such as ‘democracy’ or ‘human rights’, as the signifiers of belonging can be seen as having the most permeable boundaries of all” (Yuval-Davis, 2006: 209).

We emphasize that being granted the right to belong does not rely only on gatekeepers’ decisions. The metaphor of the gatekeeper helps express how formal membership can be granted or denied. However, it is important to note that other practices of organizations can also be mechanisms for granting or denying belonging. Mecheril (2018), for example, argues that “anticipated denial” of belonging needs to be taken into consideration as well. Other authors stress that certain practices in an organization's culture can lead to exclusion and make it more or less likely that emotional attachments develop. Examples of how belonging can be denied include lack of representation, stereotyping, assimilation pressure, and micro-aggressions (e.g. telling racist or sexist jokes) (Bradbury, 2013; Burdsey, 2011; Elling and Claringbould, 2005; Fletcher and Spracklen, 2014; Seiberth et al., 2013). On the other hand, creating a positive, welcoming environment can have a powerful effect on enabling a sense of belonging (Doidge et al., 2020).

#### Transferring the concept of belonging to research on amateur sports clubs

The concept of belonging has also been used in the sociology of sport. Academics have studied how specific sports, sports clubs or so called ethnic-specific teams (Fletcher and Walle, 2015) become reference points of identification and belonging, how requirements of belonging and symbolic boundaries in specific sports may change once players from minority ethnic backgrounds enter the game, how feelings of belonging are developed in different sports settings, and, at least to some extent, how sport and belonging are negotiated in public and political discourse (Burdsey, 2015, 2016; Fletcher and Walle, 2015; Lenneis and Agergaard, 2018; Spracklen, 2007; Spracklen and Spracklen, 2008; Walle, 2013). They have shown that joining a sports club or a team can create feelings of belonging (Burrmann et al., 2017; Lenneis et al., 2020; Walseth, 2006); that “ethnic-specific” teams and leagues can provide “an escape from everyday racism” (Fletcher and Walle, 2015: 236); that clubs can be “second families” for refugee youth (Spaaij, 2015); and that they can be a site for socialization experiences that may “cultivate a sense of belonging and reduce social isolation” (Spaaij, 2012: 1520; also see Doidge et al., 2020).

Scholars have also investigated how the development of belonging is associated with the *politics* of belonging: for example, how belonging is associated with an organization's culture (Burrmann et al., 2017; Doidge et al., 2020; Fletcher and Spracklen, 2014), with a specific policy of ensuring a safe space for marginalized groups (Lenneis et al., 2020), or with public discourse. This research shows that different marginalized groups (e.g. Black players, British Asian players in the UK, minority ethnic players) don't necessarily develop a sense of belonging once they have joined a sports club but that sport can “provide places for belonging and exclusion” (Spracklen and Spracklen, 2008: 215; also see Ratna, 2010). Clubs can also be sites for “the reproduction of white heterosexuality” (Adjepong, 2017: 218), of marginalization, of exclusion and of assimilation pressure—for example when belonging and acceptance are—as Spracklen and Spracklen (2008) have shown for minority ethnic rugby players in the north of England—bound to demonstrate “the ability to embrace a working-class, northern culture of whiteness” (p. 215; also see Burdsey, 2011; Engh et al., 2017; Fletcher and Spracklen, 2014; Massao and Fasting, 2014; Ratna, 2010, 2013; Spracklen, 2007).

Consequently, we are neither the first to address the topic of sport and belonging nor are we the first to address exclusion or discrimination in sport. However, most of the research that has been conducted so far is qualitative; it tends to focus on processes of exclusion and discrimination that appear *after* immigrants or other marginalized groups have become members of a club. The present study is different because we have chosen an earlier starting point: how permeable are the borders of a sports club in the first place? We assume that lower membership rates of first- and second-generation immigrants might, in part, be related to the aforementioned “dirty works” of boundary maintenance. By denying access to sports clubs, gatekeepers do not necessarily follow official guidance: they may decide to grant access based on common descent, race, or citizenship, and may thus contribute to the rather opaque policies of boundary-keeping. Assuming that membership often starts with a request for participation in a practice session, we can thus operationally define the gatekeepers as those who reply to such requests. In most cases, these are coaches, managers, or administrative employees of the sports clubs.

### Research design and methods

We use the publicly available data from a field experiment performed by Gomez-Gonzalez et al. (2021) to discuss in detail the implications for Germany.

**Figure 1.**
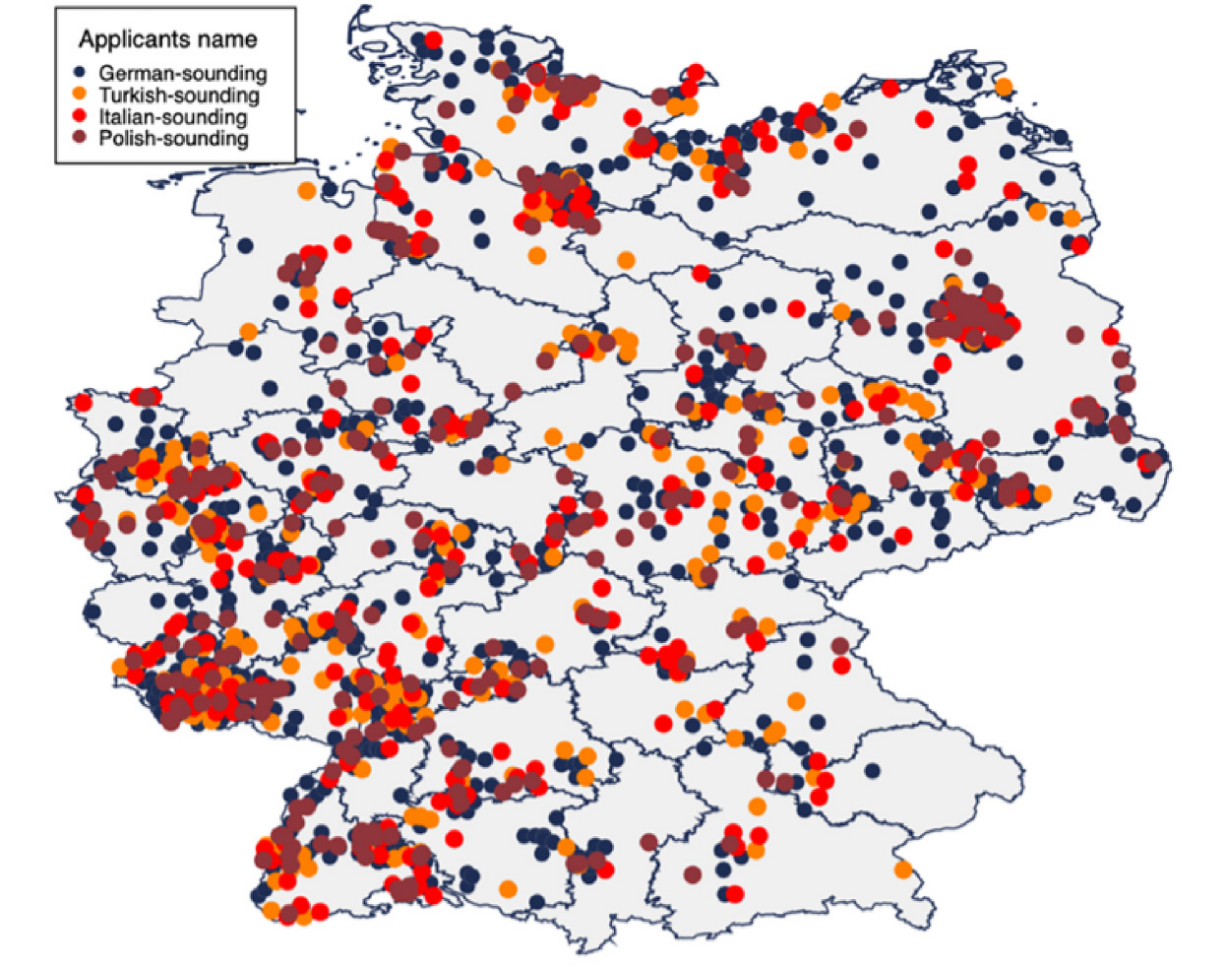
German amatuer football clubs and group name.

The experiment was set up as follows. First, information was gathered about 1,681 amateur football clubs with male teams in Germany that compete in leagues with no restrictions on foreign players. For each club, contact email addresses were identified; usually these were for the coach or an administrator. If a club had more than one team, one was randomly selected to avoid suspicion that could stem from receiving several emails with the same purpose at the same time. Focusing exclusively on male sports clubs is a shortcoming of the study. Figure 1 shows the distribution of the clubs.

Second, mock applications were sent to each of the 1,681 clubs from fake gmail.com accounts. The accounts were associated with typical foreign- and German-sounding names. The German-sounding names were Philipp Fischer, Daniel Müller, Maximilian Schmidt, Lukas Schneider, and Christian Weber. The foreign-sounding names were either Turkish (Mehmet Çelik, Mustafa Şahin), Polish (Jakub Kamiński, Mateusz Wiśniewski), or Italian (Andrea Bianchi, Francesco Esposito), as these are the three largest foreign groups in Germany (Eurostat, 2019).

Block-randomization was used at the state level, meaning that every name and every group was equally distributed within Germany (see Figure 1). In their email to the coach, the fictitious men asked whether it was possible to join a training session. The email, in grammatical German, was identical for all clubs: only the name of the requester differed. The identity of the applicant could therefore be inferred only from the name. Recipients of the email saw the name of the applicant twice: in the profile name and in the signature at the end of the message. Translated into English, the text of the email was as follows:

*Subject: Trial practice*

*Hello,*

*I would like to take part in a trial training session with your team. I have already played at a similar level. Could I come for a trial training session?*

*Many thanks*

*Name*

In total, 836 emails were sent with a German-sounding and 845 with a foreign-sounding name. Of the foreign-sounding names, 281 were Turkish, 282 Italian, and 282 Polish.

Responses from the coach (or administrator) were categorized as follows: (1) no response or rejection, (2) positive response, or (3) positive response with inquiries. We follow similar empirical field experimental papers that classify “no response” as a rejection (Agan and Starr, 2018; Barach and Horton, 2021; Edelman et al., 2017; Sawert, 2020). In the third category, additional questions related to playing position, experience, or previous clubs. To simplify the analysis, Categories 2 and 3 were combined. Thus, we used a binary dependent variable: no response or rejection (0) versus positive response (1).

The field experiment by Gomez-Gonzalez et al. (2021) received ethical approval from the University of Zurich (IRB approval #2019–006). Although deception is a necessary part of the design, it is minimized because the researchers immediately sent an email back to the respondents to the effect, “Thank you, but I’m no longer interested in playing.” Thus, respondents invest very little time in the non-existent individual. If respondents knew that the individual who applied does not exist, they would have no incentive to reply.

## Results

**Figure 2.**
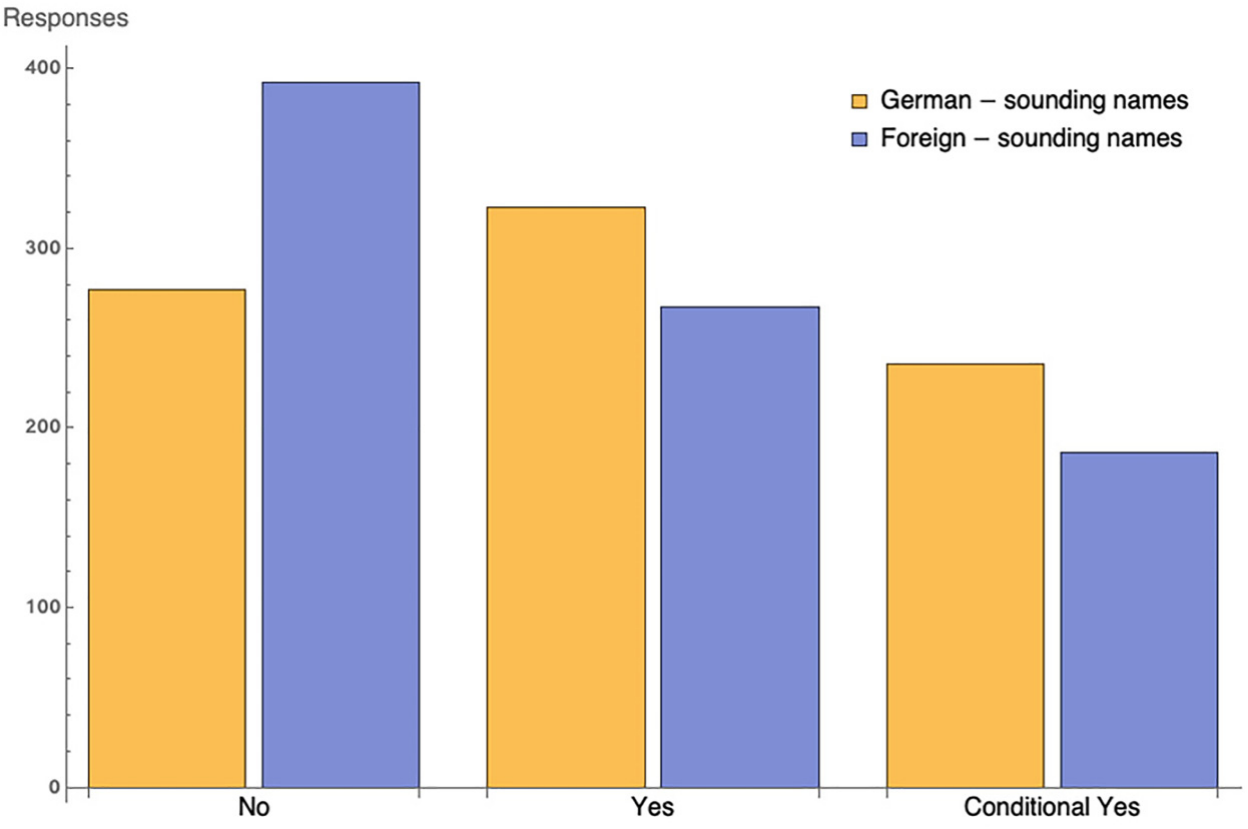
Differences in the type of response for foreign and native names.

When requesting a trial practice, 559 of 836 (66.9%) of the emails signed with German-sounding names received positive responses, compared to 453 of 845 (53.6%) emails signed with foreign-sounding names. Figure 2 shows the differences in types of response by group.

As mentioned, the mean positive response rate was 66.86% for German-sounding names, 53.61% for foreign-sounding ones (average treatment effect = 0.133; Mann-Whitney U, z =  − 5.55, p = 0.00, N = 1681). Table 1 shows the regression results for this significant difference (Model 1). Turkish-sounding names had a response rate of 55.16%, Italian-sounding 50.35%, and Polish-sounding 55.32%; differences between groups were not significant (Table 1, Model 2).

**Table 1.** Ordinary least squares regression results by name group with additional controls.

	Dependent variable: Response (0 = No/1 = Yes)			
Variables	Model 1	Model 2	Model 3	Model 4
German-sounding names	0.133*** (0.024)	0.117*** (0.034)	0.115*** (0.034)	0.130*** (0.036)
Foreign-sounding names	omitted			
Turkish-sounding names		omitted	omitted	omitted
Italian-sounding names		−0.048 (0.042)	−0.055 (0.042)	−0.036 (0.045)
Polish-sounding names		0.002 (0.042)	−0.004 (0.042)	−0.012 (0.044)
Net migration				0.005 (0.003)
Local district population / 10,000				0.001* (0.001)
Share of right-wing votes				0.002 (0.002)
League fixed effects			Yes	Yes
Constant	0.536*** (0.017)	0.552*** (0.030)	0.546*** (0.050)	0.419*** (0.069)
Observations	1,681	1,681	1,497	1,497
Adj. R-squared	0.018	0.018	0.027	0.029

Note: Robust standard errors in parentheses. ***p < 0.01, **p < 0.05, *p < 0.1.

In randomized field experiments, control variables are expected to be uncorrelated with the independent variable of interest, and thus including them should not bias the estimates (Gerber and Green, 2008). This means that additional control variables should neither modify the sign nor the significance level of the effect of foreign names on response rate. To test whether the random assignment was successful, some control variables were included that might influence the dependent variable.

Conflict theory provides a solid ground to explore the relationship between ethnic diversity (e.g. net migration) and social outcomes (Putnam, 2007). Consequently, we included the number of inhabitants living in the area to control for differences between rural and urban settings (Musterd and Ostendorf, 2013). Right-wing ideologies may influence level of discrimination against immigrants (Bale, 2008; Helbling et al., 2010), so we controlled for share of right-wing votes in the previous elections. Finally, because discrimination may be less in higher leagues due to stronger competitive pressure, league fixed effects were included to control for differences between leagues (Kalter, 2005).

The inclusion of these control variables leads to a small drop in the number of observations due to missing data. Table 1 reports the main results for the limited sample of Model 3 and the complete results with additional control variables of Model 4. We observe that the negative effect of having a foreign-sounding name remains unchanged. All control variables are insignificant with the exception of larger populations, which have a positive influence on the response rate that is significant at the 10% level.

## Discussion

The experiment showed that requests from German-sounding profiles received significantly more positive responses than from foreign-sounding ones. The response rates indicate that the scenario enacted by the experimental set-up is consistent with social reality: asking for participation in a training session via email is not the only way to initiate membership, but it is a very common way. We suggest that the response rate would have been far lower if this procedure were not part of a club's normal practice.

Although participation in sports clubs is expected to contribute to a sense of belonging (Burrmann et al., 2017; Spaaij, 2015; Walseth, 2006), our research indicates that individuals who are perceived as immigrants do not receive the same chance to benefit. The present findings support the theoretical assumption that belonging is not an individual choice alone, but depends also on being granted the right to belong. Belonging requires the desire for participation on the part of the minority and the acceptance of participation on the part of the majority (Ward et al., 2001; Wood and Waite, 2011; Yuval-Davis, 2006). Both are required.

Boundaries of football clubs might thus not be as permeable as they are often expected and reported to be. On the contrary, we found evidence that the metaphor of the gatekeeper who protects boundaries accurately describes how membership in a football club can be granted to some and denied to others. Gatekeepers’ decisions are related to the perceived heritage of the requesters. Not being invited to a training session after sending an email does not mean that individuals have no chance of becoming members of the clubs: they could still call or just show up in person for a practice. However, it is apparent that immigrants face more obstacles than do members of the non-immigrant population. Sports clubs including women and other age groups, e.g., youth and older adults, may report different results. Future research should consider examining these settings and other social activities with rooted domestic traditions (e.g. *Schützenverein*, shooting clubs).

In this study, “no response” was the most common negative outcome of a request Related field experiments report a similar result. For example, Sawert (2020) compared the invitation rate to the shared housing market in Berlin across immigrant groups (Turks, Syrians, and Americans). Of 427 no direct invitations, only 38 were direct rejections (with 3 “more information” requests). The remaining 386 were no response at all. Of course, even though “no response” is the most effortless response, various other reasons might be responsible for not responding (e.g. being too busy or not having the authority to decide).

Whatever alternative reasons for nonresponse may be, it should not differentially affect minority ethnic groups and immigrants. Because of randomization, respondents who are, say, too busy to respond should be equally distributed across groups. Thus, different reasons may influence the overall response rate—but not the differences between groups (Gerber and Green, 2008). We expect these findings will motivate future researchers to examine in greater detail the reasons amateur football clubs do not respond equally to local- and foreign-sounding names.

Additionally, there were differences in the response rate for different foreign-sounding groups. These differences were not statistically significant. However, we agree with those who argue that the *p*-value alone offers only limited evidence against a null hypothesis (Bernardi et al., 2017; McShane et al., 2019; Wasserstein and Lazar, 2016). As McShane et al. (2019) said, it deserves to be “demoted from its threshold screening role and instead, treated continuously, be considered along with currently subordinate factors (e.g. related prior evidence, plausibility of mechanism, study design and data quality, real world costs and benefits, novelty of finding, and other factors that vary by research domain) as just one among many pieces of evidence” (McShane et al., 2019: 235). Consequently, we submit that—given the study design and descriptive statistics—the differences between foreign groups are substantial enough to warrant further investigation.

The fact that response rates to Italian-sounding names were five percentage points lower than to Polish- and Turkish-sounding names is, at the very least, interesting. This finding contrasts with other field experiments in Germany, which tend to find that Turkish-sounding names face more obstacles than other nationalities (e.g. relative to Italians in car-ride selection, Liebe and Beyer, 2021; relative to Americans in the Berlin shared housing market, Sawert, 2020). We expected a similar outcome. However, the idiosyncratic characteristics of sports—in particular, the popularity of players on the German national team—might help to explain this finding. During the last decade there have been several Polish and Turkish (but not Italian) players on the German squad (e.g. Miroslav Klose, Lukas Podolski, Ilkay Gündoğan, Mesut Özil). This explanation is supported by a study demonstrating the beneficial effects of FC Liverpool's star player, Mohamed Salah, on Islamophobic prejudices in England (Alrababa’h et al., 2021).

## Conclusion

The purpose of this paper was to empirically investigate who is granted the right to access a certain social activity, namely, joining an amateur football club in Germany. In particular, we wondered if being granted the right to belong depended on being perceived as an immigrant. We used data from a field experiment in which individuals with foreign- and native-sounding names sent identical emails to amateur football clubs asking to participate in a training session. The results show that membership is at least partly a matter of being granted the right to belong. In other words, boundary-making processes are in place in football clubs: having a foreign-sounding name reduces the likelihood of being invited to a practice session.

The results of the study raise some questions regarding past and future research. Third-sector organizations, such as sports clubs, are often regarded as formally open to everybody, offering good opportunities for equal access. In some other fields—such as becoming a citizen—it is clearly more difficult to gain access. However, the criteria for citizenship and similar fields are rather transparent, whereas the decision to grant membership in a sports club is relatively opaque. In clubs, the decision to admit someone is made by an individual. In Crowley’s (1999) model, these individuals represent the “gatekeepers”: they make choices about whom to accept. These gatekeepers are not professionals but volunteers who perform the task in their leisure time; they do not have to follow protocols and they usually do not have to defend their choices. The fact that gatekeepers’ decisions are associated with the perceived heritage of newcomers, as shown here, suggests that sports clubs are far less accessible to immigrants than is often assumed.

The finding also raises questions about mainstream academic discourse. As pointed out in the Introduction, the academic discourse about the role of sports in immigrant societies is usually a positive one that focusses on sport's integrative potential. Even the fact that first- and second-generation immigrants are less likely to be members of a sports club than their non-immigrant peers does not raise questions about ethnic discrimination, but rather leads to conclusions about cultural differences or self-exclusion. The present findings, however, show that even if culture matters, even if there are self-segregation tendencies, and even if lower participation rates of immigrants interact with socioeconomic disadvantages, discrimination does occur, and it does so at an early stage. Whereas some studies show that racist micro-aggressions and assimilation pressures appear in sports clubs (e.g. Burdsey, 2011; Engh et al., 2017; Massao and Fasting, 2014) and might lead to minorities’ dropping out, the current research shows that even immigrants who want to participate are, because of their foreign-sounding name, denied access.

Furthermore, our research supports the argument that differences in participation rates between immigrants and non-immigrants do not always represent power inequalities and that similarities do not always represent social equality (Elling and Claringbould, 2005). Even if immigrants are equally involved in sports clubs as non-immigrants—and in the German case this does hold true for male adolescents (Nobis and El-Kayed, 2019)—it does not necessarily mean that there is no discrimination. Instead, it is likely that immigrants have to put more effort than others into being accepted (Dietl et al., 2020); or, as the popular saying has it, to stay in one place they have to run twice as fast.
